# Comparison of Energy Consumption Between Single Energy O-Gantry and Dual Energy C-Arm Linear Accelerator

**DOI:** 10.1016/j.adro.2025.101762

**Published:** 2025-03-14

**Authors:** Vibhor Gupta, Suresh Chaudhary, Olajide Fadare, Shilpa Senapati, Sushil Beriwal

**Affiliations:** a American Oncology Institute, India; b Varian Medical Systems, India

## Introduction

Environmental pollution in the form of high energy usage and greenhouse gas emissions (GHG) is a major contributor to climate change, as the continued modernization of processes across industries is associated with the introduction of newer technologies that consume more energy. Prior research has identified the transportation and the health care sectors to be major sources of environmental pollution.^1^^,^^2^ Some estimates attribute about 10% of the carbon footprint of health care to clinical radiology and radiation therapy (RT).^3^ The health harms of RT-associated environmental pollution have prompted calls for the adoption of environmentally sustainable practices in radiation oncology care.

Scope 2 is one of the metrics used to measure emissions and it refers to the indirect GHG resulting from the generation of purchased electricity from a utility provider. These emissions are a key component of a company's carbon footprint and are essential in the context of Environmental, Social, and Governance reporting.^4^ There have been previous reports on the energy efficiency and reduced power usage of single energy Halcyon (O-gantry) compared with dual energy C-arm linear accelerator (LINAC). This is probably because of dual-energy machines are less efficient because of the longer waveguide and added power of the steering coils, bending magnets, and larger mass to be moved during motions. The goal of this study was to further quantify the power usage and energy savings across various activities on Halcyon LINAC (O-gantry), and the impact on Scope 2 metrics and the environment.

## Methods and Materials

American Oncology Institute cancer center in Nagpur has one C-arm dual energy LINAC that has been in use since 2018. The second LINAC (O-gantry LINAC) was commissioned in January 2024 and started treating patients in February 2024. We collected data on quality assurance (QA), treatment activities, and power consumption for the period of January 2024 to June 2024 for both C-arm and an O-gantry LINACs. We categorized LINAC power consumption for both machines based on QA, treatment activities, and machine standby mode. Time taken to perform QA and treatment activities, along with time the machine was in standby mode were measured. QA activities comprised of both intensity modulated radiation therapy (IMRT) treatment plan and machine-specific QA. The average time taken for each plan QA was about 10 minutes. Machine QA was performed daily and weekly as per TG 142 protocol. The daily QA required an average of 30 minutes. For weekly QA, a structured QA process was performed on both LINACs as per TG 142, TG 151, and TRS 398 protocols and required about 6 hours.^5^, ^6^, ^7^ Treatment activities were defined as radiation fraction for each patient along with plan-specific QA time. The LINACs were kept in standby mode when not being used for any treatment or QA procedure. Each radiation fraction took about 20 minutes on the C-arm LINAC and 7 minutes on the O-gantry LINAC. Machine QA time was calculated by adding the time used in both daily and weekly QA. For machine idle time, we assumed that the LINACs were kept in standby mode when not used for treatment or QA. Treatment time and QA time were subtracted from total operational hours in a month (24 hours multiplied by number of days in the month) to get the time spent in standby mode.

Power calculations for this study were done based on actual readings from the power meter. The power meter displays consumption in kilowatt-hour, which is equivalent to 1 unit of power. Power consumption was classified into 3 categories including power usage for treatment activities (fraction delivered plus IMRT plan QA), power usage for machine QA and power usage in standby mode. The power and time consumption for QA activity was observed manually. Power consumption averaged 11.5 and 31.2 kWh/h for the O-gantry and the C-arm machine, respectively. Power usage in standby mode was calculated by observing power usage readings at the end of the day to the start of the QA activity on the following day. Power usage during treatment activities was calculated by subtracting power used in standby mode and during machine QA activities from the total power consumption, divided by the total number of treatment activities (fractions plus plan QA). In order to account for the differing patterns of use between machines and months, means and standard deviations were weighted by the number of fractions in the month.

## Results

**Figure 1:**
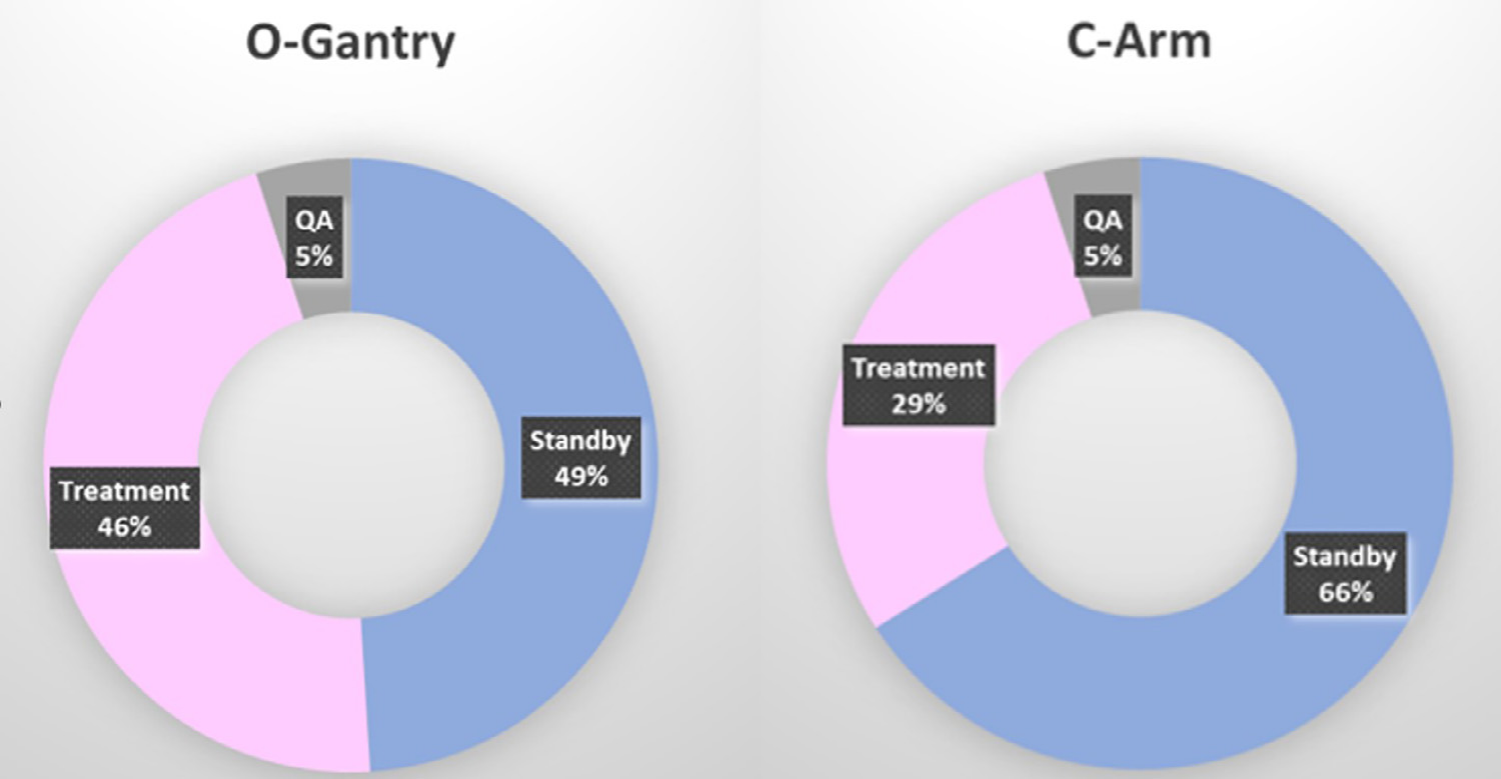
Percentage distribution of power usage across activities on the O-gantry and C-arm linear accelerators.

The number of fractions and total treatment varied across the duration of data collection (Table 1). LINAC power usage across various activities is shown in Fig. 1. For fair comparison of power usage, we chose months when the number of fractions were similar between the 2 machines (January, February, and June for C-arm, and February, March, and May for O-gantry) (see Table 1). There was no significant difference between the number of treatment activities, the treatment time, and the time spent in standby mode by both LINACs (Table 2). An average of 4 weekly machine QA activities and 21 daily machine QA activities were performed on both LINACs over the period assessed. The average duration of machine QA activities (daily plus weekly) over the period assessed was 35 hours on both LINACs. Although there was no significant difference in the number of treatments delivered on both LINACs (*P* = .85), the average power consumption of the C-arm LINAC was significantly higher than the average power consumption of the O-gantry LINAC (*P* = .004). Since technique of RT can also impact power consumption, the distribution of fraction numbers based on technique of RT was also collected (Table 3). Majority of patients had volumetric modulated arc therapy (VMAT) or IMRT on both machines with not unexpectedly more patients had 3-dimensional RT in C-arm (13% vs 3%).

**Table 1:** Activities and energy usage on C-arm and O-gantry linear accelerators based on months with similar number of fractions

			Activities and duration				Power consumption			
Machine	Month	Fractions	New start	Treatment time (h)	Standby time (h)	Treatment	Machine QA	Standby	kWh/fraction	kWh/QA
TrueBeam	January	1603	89	418.50	290.50	10,098.00	1092.00	4357.50	5.97	42.00
Halcyon	March	1584	80	337.50	277.50	2611.50	379.50	2220.00	1.57	17.25
TrueBeam	June	1057	40	291.67	393.83	9887.10	1076.40	5907.50	9.01	43.06
Halcyon	May	1193	61	351.50	357.00	3396.75	408.25	2856.00	2.71	15.12
TrueBeam	February	755	16	241.00	396.50	6444.10	1076.40	5947.50	8.36	43.06
Halcyon	February	753	103	231.83	429.67	1376.92	396.75	3437.33	1.61	15.87

*Abbreviation:* QA = quality assurance.

**Table 2:** Activities, duration, and power consumption of C-arm and O-gantry LINACs

	C-arm LINAC		O-gantry LINAC		
	Mean	SD	Mean	SD	*P*
Activity and time					
Treatment (fraction + plan QA)	1304.22	383.12	1353.10	315.33	.88
Treatment time (h)	340.00	76.05	319.69	46.17	.72
Standby time (h)	345.92	52.14	336.83	59.64	.20
Power consumption					
Treatment (fraction + plan QA)	9224.91	1484.52	2613.50	730.53	.006⁎
Machine QA	1083.72	7.79	392.90	12.78	<.001⁎
Standby	5188.77	782.11	2694.60	477.09	.02⁎
Total	15497.40	1223.16	5701.00	685.97	.001⁎
kWh/treatment	7.44	1.57	1.96	0.53	.01⁎
kWh/QA	42.56	0.53	16.24	0.95	<.001⁎

*Abbreviations:* LINAC = linear accelerator; QA = quality assurance.

⁎ Significant difference P<0.05.

**Table 3:** Distribution of fraction numbers based on technique of radiation

	C-arm	O-gantry	C-arm	O-gantry	C-arm	O-gantry
Month	January	March	June	June	Total	Total
3D CRT	171	82	73	8	244	90
IMRT	638	475	457	255	1094	730
SBRT	14	20	13	5	27	25
VMAT	870	1088	551	754	1421	1842
Electron	-		3		3	-
Grand Total	1692	1664	1097	1022	2789	2686

*Abbreviation:* 3D CRT = three-dimensional conformal radiation therapy, IMRT = intensity modulated radiation therapy, SBRT = stereotactic body radiation therapy and VMAT = volumetric modulated arc therapy.

Significantly lower energy was consumed by the O-gantry LINAC for machine QA (mean = 395 kWh) compared with the C-arm LINAC (mean = 1081 kWh). Similarly, the O-gantry LINAC used significantly lesser energy (mean = 2838 kWh) in standby mode than the C-arm LINAC (mean = 5405 kWh). The largest percentage decrease in power consumption between the C-arm LINAC and the O-gantry LINAC was observed in the power usage for treatment delivery (72.06%). The percentage decrease in power usage for machine QA was 63.5% and was 44.49% for standby (see Table 2).

## Discussion

Health care constitutes about 10% of GHG emission and any reduction in emission can help reduce the impact of health care on global climate change. Radiation therapy machines and treatment are a significant part of power consumption in hospitals. In one study the GHG emission associated with a typical external beam RT cancer treatment was estimated to range from 185 to 2066 kg CO_2_eq, with about 38% of these values determined to be related to LINAC acquisition and maintenance.^8^ Our study shows that the single energy O-gantry LINAC saved ∼6 kWh of power on each treatment delivered compared with the dual energy C-arm LINAC. In proportionate terms, this corresponds to 75% decrease in energy usage based on the case load in the unit, which represents a significant energy savings considering the lifetime capacity of the machine to deliver around 200,000 fractions. The carbon footprint of India's power sector is around 0.82 t CO_2_/MWh, which translates into reduction of about 700 tons or more of CO_2_ emissions in the life cycle of the machine.^4^ In other words, this decarbonization is equivalent to saving GHGs by burning 70,000 gallons of diesel^9^ or CO_2_ absorbed by 32,000 trees in a year.^10^

The adoption of an energy-efficient machine design by the single energy O-gantry manufacturer as an approach to reducing the carbon footprint of RT is consistent with previous studies that have described the incorporation of energy sustainability into machine design and manufacturing as a carbon footprint mitigation strategy. The power savings observed at American Oncology Institute, Nagpur following the commissioning of the O-gantry LINAC was achieved without any compromise in patient care. Treatment plans on the O-gantry were as conformal as with any other LINACs, which in combination with the mandatory inbuilt image-guided radiation therapy (IGRT) and fast treatment delivery capabilities of the O-gantry helped clinicians provide excellent care to the patients.

The carbon footprint of radiation oncology is also influenced by carbon emissions associated with the construction of health care buildings, patient and clinician transportation to clinic sites, energy usage from computers and ancillary office equipment, as well as medical supplies and waste disposal.^11^ There was additional reduction in carbon footprint with O-gantry from construction of bunker, which was not calculated for this study.

The findings of this study should be interpreted with consideration for study limitations. There are other factors that impact power consumptions that were not included in this study. For example, the specific mode of treatment, fractionation size, and photon energy can influence power usage.^12^ However, it was reasonable to assume that variations in power consumption from treatment mode and fractionation size would be similar in both machines because both LINACs were part of same practice. The use of high energy was limited as majority of patients were treated with IMRT/VMAT using 6 MV photons. The degree of power saving may be different in centers with higher percentage of patients treated with high energy and different techniques. Also, the imaging protocol used for treatment guidance (IGRT) can influence LINAC power consumption. On the O-gantry LINAC, cone beam computed tomography imaging is a mandatory part of the workflow, whereas on C-arm LINAC, cone beam computed tomography imaging can be skipped. Physicians and physicists decided the imaging protocol for each patient on the C-arm LINAC, but we did not capture that information in our analysis. To minimize variations because of number of fractions of treatments delivered, we used data for the months when fraction numbers were similar for both LINACs to compare their power consumption. The strength of the study is a detailed analysis and break down of power consumption based on activities, which gives readers more comprehensive information on the energy efficiency of the O-gantry LINAC relative to the C-arm LINAC, and the potential impact of the O-gantry on GHG emission reduction, in comparison to any other published data.

## Conclusions

The addition of the single energy O-gantry LINAC contributed to significant energy savings at our center. Although the initial motivation for acquiring the O-gantry LINAC was to have the O-gantry as a second machine along with the C-arm, or a solo machine based on the clinical need in most of our network sites, the energy savings proffered by the single energy O-gantry, combined with the faster treatment delivery of IMRT/IGRT plans allow us to achieve our goal of treating more patients and reducing wait times in our network sites in a sustainable way.

## Disclosures

The American Oncology Institute where this study was performed is owned by Varian Medical Systems. All authors employed by Varian Medical Systems. Sushil Beriwal reports consulting or advisory relationship with Elsevier Inc.
